# A Novel High-Precision Digital Tunneling Magnetic Resistance-Type Sensor for the Nanosatellites’ Space Application

**DOI:** 10.3390/mi9030121

**Published:** 2018-03-09

**Authors:** Xiangyu Li, Jianping Hu, Weiping Chen, Liang Yin, Xiaowei Liu

**Affiliations:** 1 Faculty of Information Science and Technology, Ningbo University, Ningbo 315211, China; lixiangyu7410@sina.com; 2 MEMS Center, Harbin Institute of Technology, Harbin 150001, China; weipingchen1@outlook.com (W.C.); 15B921019@hit.edu.cn (L.Y.); liuxiaowei3@outlook.com (X.L.)

**Keywords:** MEMS, interface circuit, chopper instrumentation amplifier, Sigma-Delta

## Abstract

Micro-electromechanical system (MEMS) magnetic sensors are widely used in the nanosatellites field. We proposed a novel high-precision miniaturized three-axis digital tunneling magnetic resistance-type (TMR) sensor. The design of the three-axis digital magnetic sensor includes a low-noise sensitive element and high-performance interface circuit. The TMR sensor element can achieve a background noise of 150 pT/Hz^1/2^ by the vertical modulation film at a modulation frequency of 5 kHz. The interface circuit is mainly composed of an analog front-end current feedback instrumentation amplifier (CFIA) with chopper structure and a fully differential 4th-order Sigma-Delta (ΣΔ) analog to digital converter (ADC). The low-frequency 1/*f* noise of the TMR magnetic sensor are reduced by the input-stage and system-stage chopper. The dynamic element matching (DEM) is applied to average out the mismatch between the input and feedback transconductor so as to improve the gain accuracy and gain drift. The digital output is achieved by a switched-capacitor ΣΔ ADC. The interface circuit is implemented by a 0.35 μm CMOS technology. The performance test of the TMR magnetic sensor system shows that: at a 5 V operating voltage, the sensor can achieve a power consumption of 120 mW, a full scale of ±1 Guass, a bias error of 0.01% full scale (FS), a nonlinearity of *x*-axis 0.13% FS, *y*-axis 0.11% FS, *z*-axis 0.15% FS and a noise density of *x*-axis 250 pT/Hz^1/2^ (at 1 Hz), *y*-axis 240 pT/Hz^1/2^ (at 1 Hz), *z*-axis 250 pT/Hz^1/2^ (at 1 Hz), respectively. This work has a less power consumption, a smaller size, and higher resolution than other miniaturized magnetometers by comparison.

## 1. Introduction

**Figure 1:**
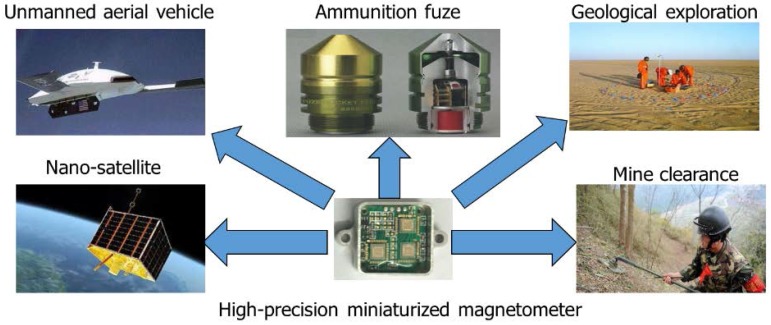
The high-precision miniaturized magnetometer in military and civilian field.

The earth is a huge magnetic source and scatters around the weak magnetic field (about 50 μT). So, there is a specific relationship between the size, direction of the magnetic field, and geographical position. We can achieve the high-precision GPS navigation by obtaining accurate geomagnetic field information. It is strategic and tactical for concealed combat equipment (such as submarines, stealth aircraft, etc.) [1]. The ferromagnetic objects (such as ore, magnetic conducting metal, armored vehicles, warships, submarines, etc.) can change the distribution of the geomagnetic field and generate anomaly magnetic field. If we can accurately measure the anomaly magnetic field, we can get the location, size, and other information of the target object [2]. As shown in Figure 1, the magnetic detection system plays an important role in the nano-satellite, unmanned aerial vehicle antisubmarine, ammunition fuze, geological exploration, mine clearance, and traffic monitoring. In the geomagnetic field detection, the signal amplitude of geomagnetic field and anomaly magnetic field are very weak, the signal frequency is very low (about 1 Hz).

Miniaturization high-performance magnetometers based on AMR (anisotropic magnetic resistance), GMR (giant magnetic resistance), and TMR (tunneling magnetic resistance) are widely used in military and civilian field. It is difficult to improve the change rate of magneto-resistance based on the principle of anisotropic scattering. TMR based on tunneling current has a lager change rate of magneto-resistance compared with GMR and AMR. The TMR magnetometer has a higher sensitivity and a wider linear range as the third generation magneto-resistance sensor, but it has a lager noise at low-frequency than GMR and AMR because of tunneling effect. At present, the research on TMR sensors still stays on the study of sensitive surface materials. The research on reducing 1/*f* noise of TMR sensor and digital interface Application Specific Integrated Circuit (ASIC) for TMR sensors has not been reported. The study of a digital magnetometer based on TMR effect is significant. In the nanosatellites’ space application field, magnetic sensors are widely used for attitude control. Because of miniaturization and low power consumption the magnetic resistance-type sensor is selected in most nanosatellites [3]. Magnetic sensors based on very large scale integration (VLSI) can combine with MEMS magnetic sensitive element. Honeywell manufactures several sensors based on AMR: one-axis HMC1021, two-axis sensors (HMC1022), and three-axis analog output (HMC1043) and digital output (HMR2300) with an integrated Application Specific Integrated Circuit (ASIC) [4,5,6]. For example, in 2004, the ION-F (Ionospheric Observation Nanosatellie Formation) mission consists of three nanosatellites that were built at Utah State University and University of Washington. The four one-axis HMC1021 sensors are used for the attitude determination in NANOSAT-01 and NANOSAT-1B. This sensor has a linearity error of 0.4% FS, a noise density of 48 nV/Hz^1/2^ and a sensitivity of 1 mV/V/Guass [7]. In 2009, the three-axis HMC1043 was applied in Spanish OPTOS satellite. It has a resolution of 13 nT and a noise density of 50 nV/Hz^1/2^. The power consumption is increased to a total number of 500 mW.

**Figure 2:**
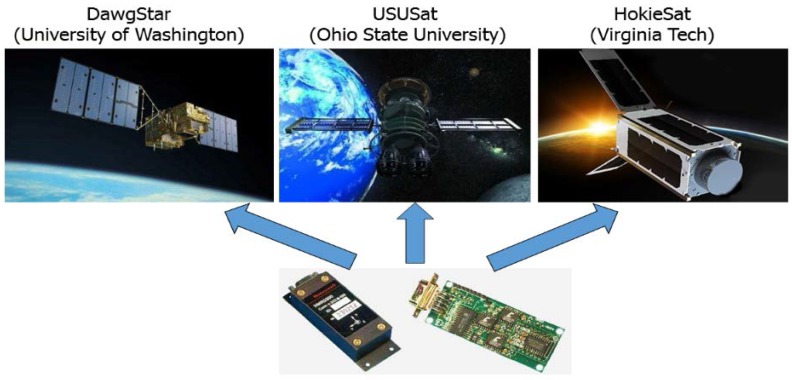
The HMR2300 magnetometer in Nano-satellite field.

As shown in Figure 2, the nano-satellites applied the Honeywell HMR2300 sensor: DawgStar satellite (University of Washington, Seattle, WA, USA), USUSat (Ohio State University, Columbus, OH, USA) and HokieSat (Virginia Tech, Blacksburg, VA, USA) [8]. The three-axis magnetometer HMR2300 with digital output can directly communicate with the computer. Three-axis independent structure can directionally detect the magnetic field of *x*-axis, *y*-axis, *z*-axis. The change voltage signal is amplified by the front-stage amplifier and converted to the digital signal by a 16-bit ADC. The sampling rate of input data, output format, average reading, and zero-bias offset can be set. The measure range is up to ±2 Gauss; the resolution is 6.7 nT; the sampling speed is 10–157 sampling points/s the three-axis digital output is BCD or binary code; the baud rate can be chosen 9600 or 19,200; the serial port can be chosen standard RS485 or RS232 for single-point reading; the volume is 70 × 37 × 24 mm^3^; and, the power consumption is 400 mW. It has a higher resolution and a better linearity error of 0.1–0.5% FS, but it has a big volume because of detection circuit and digital processing circuit [9,10]. Because of low sensitivity and linearity based on AMR and low-performance detection circuit in HMR2300, the precision of the magnetometer cannot be improved. But, in TMR sensors, the 1/*f* noise problem should be solved. The 1/*f* noise that is caused by a magnetic mechanism is always an important factor in affecting low-frequency magnetic detection capability of TMR sensors. According to the recently domestic and foreign research reports, high-frequency modulation method based on magnetic signal is the majority method to reduce 1/*f* noise in the miniaturized magnetoresistive sensor. In most of the micro/nano-satellite satellites miniaturization magnetic sensors are equipped with discrete devices, which have large volume and high power consumption. According to previous studies, the researchers used MgO materials as the barrier layer to improve the sensitivity of the TMR sensor, but the low-frequency noise cannot be reduced. The periodic modulation of the magnetic elements in the sensor at the chopping frequency have been proposed and tested for AMR and GMR. Here, we investigate the application of chopping techniques for TMR magnetic elements. The research on high performance digital interface ASIC for TMR sensors has not been reported. The study of high-precision miniaturized three-axis digital magnetic sensor system is necessary and significant.

In this paper, we propose a high-precision miniaturized three-axis digital TMR magnetic sensor for nanosatellites’ space application. The TMR sensor element can achieve a background noise of 150 pT/Hz^1/2^ by the vertical modulation film. The analog front-end interface and digital ASIC chip for the tunneling magneto-resistive sensor are implemented by 0.35 μm mixed signal 5 V CMOS technology. The performance test of the TMR magnetic sensor system shows that: lower power consumption (120 mW), higher resolution (250 pT/Hz^1/2^ (at 1 Hz)), better linearity (lower than 0.2% FS), and smaller volume (25 × 25 × 10 mm^3^) than other miniaturized magneto-resistance sensor.

The tunneling magneto-resistance sensor element, current feedback instrumentation amplifier with chopper technique, Sigma-Delta modulator and digital decimation filter are introduced and designed in Section 2. In Section 3, we show a vertical modulation film structure and a detailed ASIC interface circuit. The performance can be improved by chopper technique, offset reduction loop technique, and dynamic element matching. The performance parameters of ASIC are tested by the experiments. Finally, Section 4 concludes the study of MEMS/TMR three-axis integrated magnetometer prototype and testing results, which shows that the performance of miniaturized TMR digital magnetometer has great advantages in the application of nano-satellite field.

## 2. Materials and Methods

### 2.1. Materials

The tunneling magneto-resistance sensor element with the multilaminar structure is from Multidimension Technologies (Suzhou, China). The TMR sensor interface circuit is fabricated by 0.35 μm CMOS process and cooperated with Shanghai Huahong Integrated Circuit (Shanghai, China).

### 2.2. Tunneling Magnetic Resistance-Type Sensor Element

**Figure 3:**
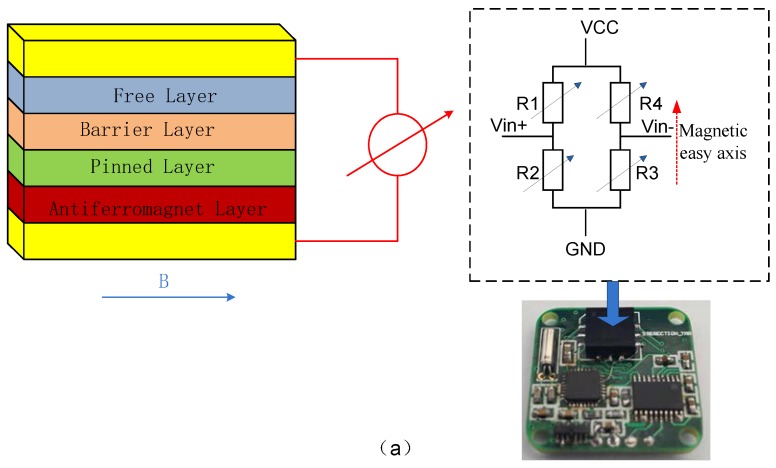

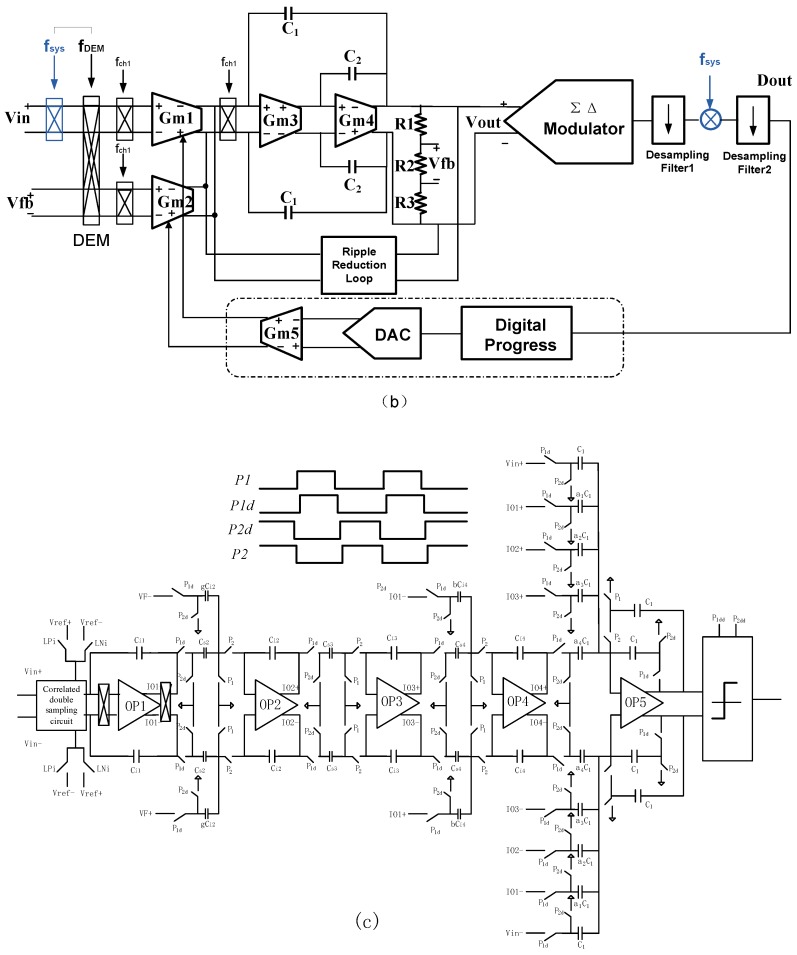
(**a**) Tunneling magnetic resistance-type sensitive structure, the physical printed circuit board (PCB) diagram is underneath the blue arrow (**b**) Three-axis digital tunneling magnetic resistance-type (TMR) sensor interface circuit System block diagram, the closed-loop feedback control of digital progress is shown in the dotted box (**c**) The fully differential 4th-order Sigma-Delta modulator.

The sensitive structure part of tunneling magneto-resistive sensor mainly consists of Pinning Layer, Tunnel Barrier, and Free Layer. The pinning layer is composed of a ferromagnetic layer and an anti-ferromagnetic layer (AFM Layer). The exchange coupling between the ferromagnetic layer and the anti-ferromagnetic layer determines the direction of the magnetic moment of the ferromagnetic layer; the tunneling barrier layer is usually composed of MgO or Al_2_O_3_, located in the upper part of the anti-ferromagnetic layer [11]. As shown in Figure 3a, the arrows represent the direction of the magnetic moment of the pinning layer and the free layer. The magnetic moment of the pinning layer is relatively fixed under the action of the magnetic field. The magnetic moment of the free layer is relatively free and rotatable to the magnetic moment of the pinning layer, and it will turn over with the change of the magnetic field. The typical thickness of each film layer is between 0.1 and 10 nm [12]. The magnetic sensor system is mainly composed of magnetic resistance-type sensitive element and CMOS readout integrated circuit. The sensitive element concludes 32 magnetic tunneling junctions (MTJ). The unit area resistance value RA of the magnetic tunneling junction is 2.5 kΩ/μm^2^, the area of magnetic tunneling junctions is 50 μm^2^. In this paper, the thickness of free layer/barrier layer/pinning layer is 10/1/10 nm. The multilayer structure of MTJ is Ta/Ru/Ta/PtMn/CoFe/Ru/CoFeB/MgO/CoFeB/NiFe/Ru/Ta, which structure is cooperated with Multidimension Technology Company. The thin film is deposited by magnetron sputtering. The MgO material is used as the barrier layer so that TMR element is more sensitive and higher resolution [13,14,15,16]. The Wheatstone bridge configuration is composed of four active TMR arrays that are applied by the thin film process, as shown in Figure 3a. The three-axis TMR sensitive element is built by stereoscopic orthogonal package.

### 2.3. Instrumentation Amplifier with Chopper Technique

The output signal of TMR sensors is at a low-frequency (about 1 Hz) and a millivolt range. Therefore, they need amplifiers to boost such a signal to be compatible with the input ranges of Sigma-Delta Analog-to-Digital Converters. Although the differential output voltage signal of the TMR sensors (V_in_) can be as small as a few millivolts, the common-mode (CM) voltage V_CM_ depending on the application can be much larger and even vary at the range of a few volts during the operation, as shown in Figure 3b. To accommodate this variable CM voltage, an Instrumentation Amplifier is generally used for the read-out circuit of the sensors [17,18,19]. To accurately process the millivolt-level signal of the TMR sensor, the input referred error of the current feedback instrumentation amplifier (CFIA) should be at the microvolt or nanovolt-level [20,21,22,23,24]. To reduce 1/*f* noise, the chopper technique is applied in the circuit. To cope with varied CM voltage, the CFIA should have a common-mode rejection ratio (CMRR) greater than 120 dB. Furthermore, this CFIA is critical since it determines the overall performance of the read-out IC. To sum up, the main functions of this amplifier:

- Amplify the weak differential voltage (V_in_);
- Low offset, low noise and low corner frequency (<5 mHz);
- High common-mode rejection ratio (>120 dB); and,
- High input impedance for TMR sensor.

### 2.4. Sigma-Delta Modulator and Digital Decimation Filter

Figure 3b shows the ASIC part of the TMR magnetic sensor system. The output of the CFIA is digitized by a fully differential 4th-order Sigma-Delta ADC, which consists of a ΣΔ modulator and a decimation filter [25,26,27]. The circuit structure and sequence diagram of the fully differential modulator is as shown in Figure 3c. In order to achieve low noise and low offset, the chopper and correlated double sampling technique are both applied in the first stage integrator. The modulator can achieve a better noise suppression performance at low frequency [28,29]. The one-bit quantizer is achieved by the dynamic comparator. The output of the comparator is as a control signal to control feedback reference voltage V_ref+_ and V_ref−_ in the first stage integrator. As shown in Figure 3c, wherein P1 and P2 are the two-phase non-overlapping clock, P1 is active-high, P2 is active-low. The shutdown time of P1d is later than P1, The shutdown time of P2d is later than P2, it can effectively suppress the influence of charge injection and clock-feedthrough in the switched-capacitor circuit. The fully differential structure can effectively suppress even harmonics of ΣΔ ADC [30]. 

## 3. Result and Discussion

### 3.1. Noise Matching

**Figure 4:**
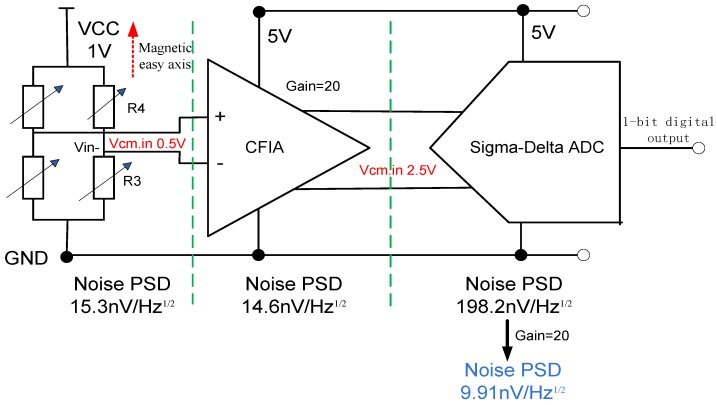
The noise performance of TMR sensor element, instrumentation amplifier and Sigma-Delta modulator.

Noise matching between the TMR sensor element, the instrumentation amplifier and the ΣΔ modulator is important effect on the precision of the TMR sensor system. The CFIA is critical in the interface circuit since it mainly determines the overall noise performance. The equivalent input noise of CFIA should be less than or equal to the TMR sensor element. To maintain the signal to noise ratio (SNR) of CFIA, the target resolution of the Sigma-Delta ADC is more than 18 bits and the background noise is less than −140 dB. The power spectrum density of each part is shown in Figure 4. The closed-loop gain of CFIA is 26 dB.

### 3.2. Noise Characteristics of CFIA with TMR Sensor Element

**Figure 5:**
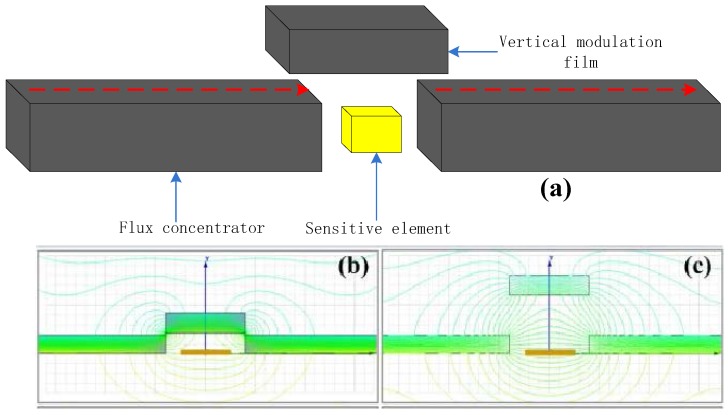
(**a**) The flux modulation micro-structure (**b**) TMR sensor with flux concentrators and choppers at OFF position (**c**) The choppers at ON position, the green line represents the size and direction of the magnetic field in the simulation.

The 1/*f* noise caused by magnetic mechanism is always an important factor in affecting low-frequency magnetic detection capability of TMR sensor. Here, we used the high frequency modulation method based on magnetic signal to reduce the low frequency 1/*f* noise of TMR sensor element. The flux modulation structure is as shown in Figure 5. The TMR sensor element is achieved to modulate by the vertical modulation film. The vertical modulation film based on the dynamic magnetic signal idea can directly modulate the measured magnetic field by the high-frequency vibratory machine-electric-magnetic microstructures. When the vertical modulation film is close to the TMR sensitivity element, the magnetic line of force is easier to pass through the vertical modulation film. The magnetic field will rapidly weaken near the TMR sensitivity element, as shown in Figure 5b. On the other hand, the vertical modulation film is far away from the TMR sensitivity element. The magnetic line of force tends to pass through the TMR sensitivity element. The shunting action of the vertical modulation film will decrease and the magnetic field near the TMR sensitivity element will be restored. Therefore, when the driving structure is in the case of high-frequency vibration, the periodic high-frequency vibration of the vertical modulation film is accompanied by the driving structure. The magnetic field near the TMR sensitivity element will be modulated. The TMR sensitivity element can detect an alternating magnetic field at this time because of the periodic high-frequency vibration. The finite element simulation of magnetic field with the vertical modulation film is shown in Figure 5b,c. The TMR sensor element can achieve a background noise of 150 pT/Hz^1/2^ at a modulation frequency of 5 kHz.

### 3.3. Noise Characteristics of CFIA with Chopper Technique

Because the low-frequency 1/*f* noise of TMR sensors is the main noise, we use the vertical modulation film structure to reduce 1/*f* noise of the TMR element. But, in the circuit the 1/*f* noise of CFIA is large at low-frequency. This chopper method is effective. The relationship between the cutoff frequency of the chopper constituted of the analog switch and the input capacitance:

(1) $$\frac{1}{2\pi R_{S}C_{G}}=\frac{1}{2\pi \mu_{n}C_{ox}C_{G}\frac{W}{L}\left(V_{GS}-V_{TH}\right)}=8f_{chop}$$

After the voltage noise of the analog switch is modulated by chopper-stabilized. The high frequency (*f_chop_*) noise is modulated to low frequency, the equivalent input voltage noise density can be shown:

(2) $${\bar{V}}_{n}(f)\approx \mu_{n}C_{ox}\frac{W}{16\pi^{2}LC_{G}(f-f_{chop})}\sqrt{\frac{K_{f}}{WLC_{OX}(f-f_{chop})}}\cdot V_{OS}$$

(3) $$\frac{W}{L}=\frac{1}{16\pi \mu_{n}C_{ox}C_{G}\left(V_{VCC}-V_{in+}-V_{TH}\right)f_{chop}}$$

**Figure 6:**
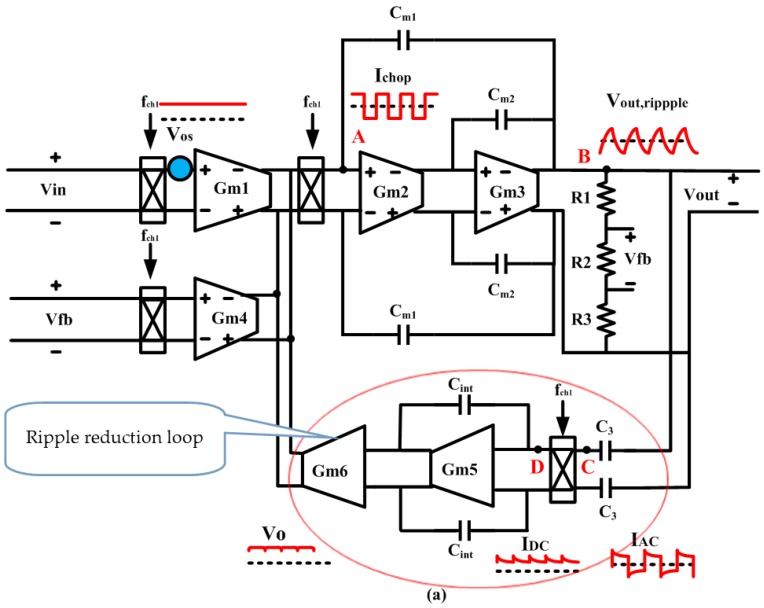

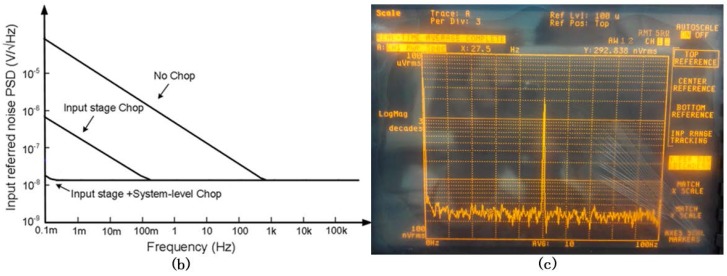
(**a**) Current feedback instrumentation amplifier (CFIA) circuit structure with ripple reduction loop (**b**) Simulating noise spectrum of with various noise chopper techniques (**c**) The power spectrum noise of CFIA.

Equations (2) and (3) shows that the relationship between the voltage noise density of analog switches, the chopper stabilized amplifier’s input offset voltage and the size of analog switches. At the same time, the minimum area of the analog switch is limited. In terms of tunneling magneto-resistive sensors’ weak signal, the 1/*f* noise and KT/C noise are considered in the design of TMR interface ASIC [31,32,33]. In order to suppress the excessive noise, high-voltage CMOS technology of the high-amplitude clock feed-through and charge injection circuit are applied. In addition, the factors affecting the noise characteristics of the chopper switches are as below: charge leakage, parasitic capacitance, IC substrate coupling noise, voltage stability of the drive signal, and the external electric field sensitive electrodes [34,35]. The factors have been considered and optimization. In order to further reduce offset, system-level chopper can also suppress the CFIA’s 1/*f* noise. The 1/*f* noise corner frequency is reduced from 10 to 0.3 Hz by the input-stage chopper. As long as the system-level chopper frequency is more than 10 kHz, the residual 1/*f* noise is suppressed. The simulating noise spectrum of various 1/*f* noise suppression techniques is shown as Figure 6b. The combination of the input-stage chopper and system-level chopper can achieve the best 1/*f* noise characteristics in the interface circuit. This front-end readout circuit can achieve mHz-level corner frequency and nanovolt-level offset. To improve gain accuracy, the dynamic element matching (DEM) is used for averaging out the mismatch between the input and feedback transconductors. The input-stage chopper frequency is 30 kHz, the resulting ripple is suppressed by a continuous-time ripple reduction loop (RRL), as shown in Figure 6a. The spectrum analyzer HP35670A (Hewlett-Packard, Palo Artaud, CA, USA) shows that the closed-loop gain is 26 dB, the unit gain bandwidth is 50 kHz (the signal bandwidth is 0–1 kHz), and the equivalent input noise density is 14.6 nV/Hz^1/2^, as shown in Figure 6c.

The corner frequency of operational amplifier 1/*f* noise is usually about 10 kHz. To achieve a corner frequency of 1 mHz, the chopper technology of input-stage amplifier is applied. Meanwhile, the corner frequency of the second-stage equivalent to the first-stage should also be less than 1 mHz. Therefore, the input-stage DC gain *A*_01_ is required:

(4) $$A_{01}\geq 20\;\log_{10}\frac{10\;\mathrm{kHz}}{1\;\mathrm{mHz}}=140\;\mathrm{dB}$$

**Figure 7:**
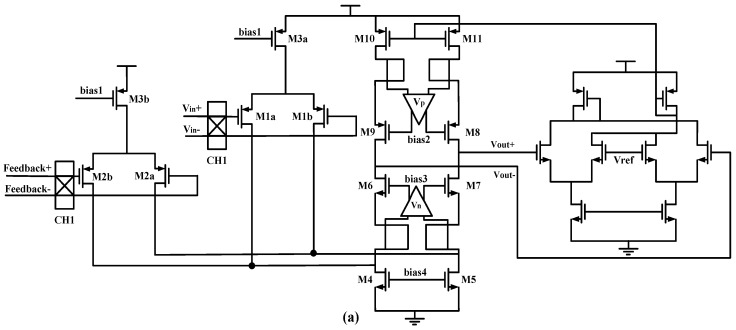

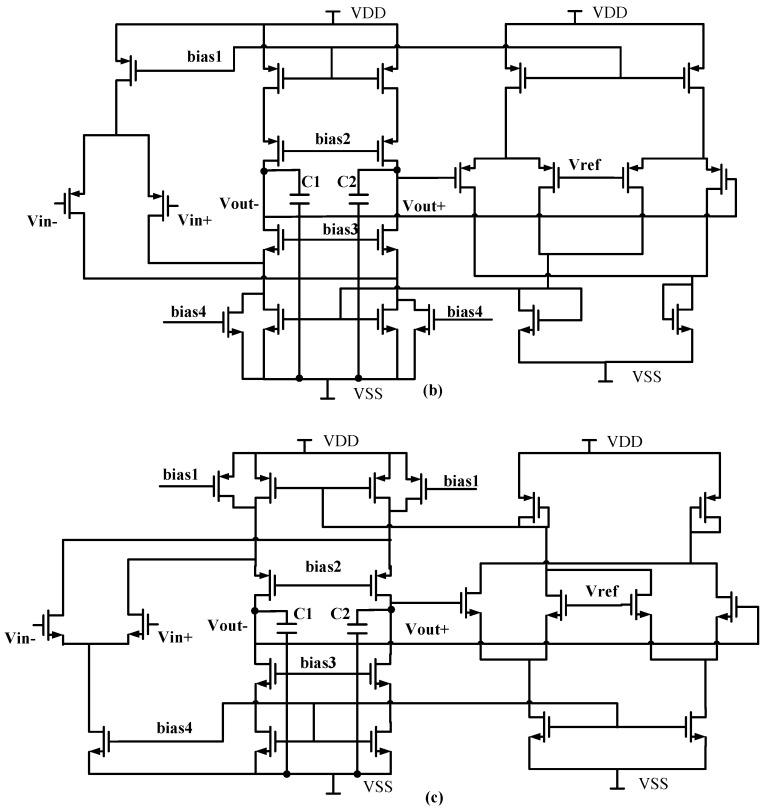
(**a**) Input stage transconductance operational amplifier with common-mode feedback circuit (**b**) Gain booster V_n_; and, (**c**) Gain booster V_p_.

At the same time, the larger DC gain can effectively suppress the noise and the nonlinearity of the post-stage. The design of input-stage transconductance Gm1 and the current feedback transconductance Gm2 is particularly important, which determines the overall performance of the CFIA. To get higher gain accuracy, the circuit and layout are designed to satisfy the size and symmetry. The circuit structure of the input-stage chopper amplifier is shown in Figure 7a. A fully differential folded cascode structure with common-mode feedback is applied in the input-stage circuit. The circuit uses gain-booster (V_n_ and V_p_) technology to improve the gain, and the circuit structure of V_n_ and V_p_ is shown in Figure 7b,c.

### 3.4. Noise Characteristics of Sigma-Delta Modulator

**Figure 8:**
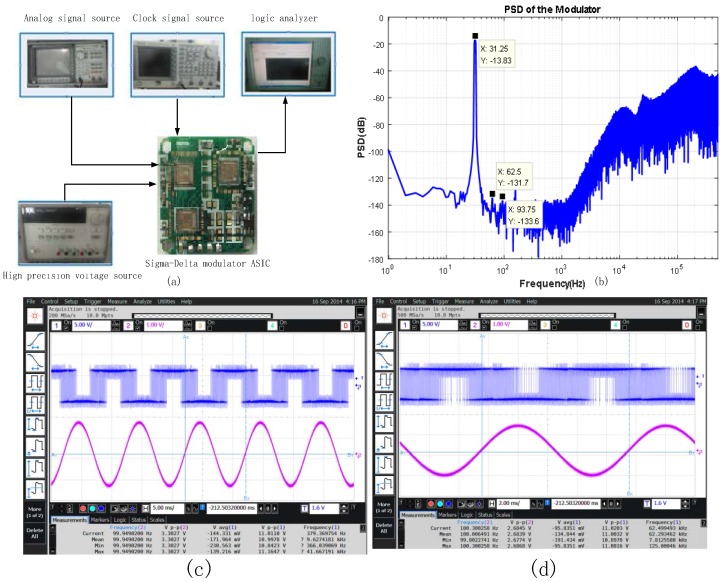
(**a**) The testing system of Sigma-Delta modulator Application Specific Integrated Circuit (ASIC); (**b**) The PSD of Sigma-Delta modulator; (**c**) The transient response of Sigma-Delta modulator; and, (**d**) Transient response after local amplification.

In order to maintain the SNR of CFIA, the noise performance of the Sigma-Delta modulator at an over sampling ratio (OSR) of 128 is as shown in Figure 4. The target performance of Sigma-Delta modulator: a background noise of less than −140 dB within a 1 kHz signal bandwidth; a SNR of more than 110 dB; an effective number of bits more than 18 bits; a harmonic distortion of less than −110 dB. To obtain a conversion time less than 0.2 s, the required sampling frequency is only 30 kHz, which is equal to the chopper frequency of the CFIA. In order to test the noise characteristics of Sigma-Delta modulator, the Sigma-Delta modulator is implemented by standard 0.35 μm CMOS process. The testing system of the Sigma-Delta modulator is as shown in Figure 8a in this paper. The 5 V power supply is provided by Agilent 3631A (Agilent Technologies Inc, Santa Clara, CA, USA); a clock control signal is provided by the Tektronix AFG3102 function signal generator (Tek Technology Company, Shanghai, China); the analog input signal is provided by the Agilent 35670 (Agilent Technologies Inc, Santa Clara, CA, USA); the digital output signal is collected by the logic analyzer Agilent 16804A (Agilent Technologies Inc, Santa Clara, CA, USA), and then the power spectral density (PSD) analysis of the Sigma-Delta is implemented by FFT of Matlab (R2016a, MathWorks, Natick, MA, USA).

The sampling frequency is 1 MHz and the Sigma-Delta modulator bandwidth is 3.9 kHz. The input signal with a frequency of 30 Hz and an amplitude of −13 dB full scale is provided by the analog signal source. The output bit flow (65,536 points) of the designed Sigma-Delta modulator is collected by the Agilent logic analyzer 16804A (Agilent Technologies Inc, Santa Clara, CA, USA) for FFT transformation, and the output spectrum of Sigma-Delta modulator is as shown in Figure 8b. The test results show that the modulator can achieve a dynamic range of 118 dB, a SNR of 115 dB; an effective number of bits of 18.8 bits; a harmonic distortion of −110 dB in the signal bandwidth; an output background noise of −141 dB; the design of reference voltage is 2.5 V, so that the signal bandwidth equivalent input noise voltage density is 198.6 nV/Hz^1/2^. 

We verify the function of the modulator before testing the performance. The digital bit stream output is collected from the Sigma-Delta modulator by the oscilloscope Agilent MSO9104A (Agilent Technologies Inc, Santa Clara, CA, USA). The transient response of the modulator is as shown in Figure 8c,d. The input signal, the clock signal, and the digital output signal are in turn in the Figure 8c,d. From the transient waveform we can get that the modulator can achieve analog digital conversion function. We can verify the correctness of its function from the test results.

### 3.5. Design of Digital Decimation Filter

**Figure 9:**
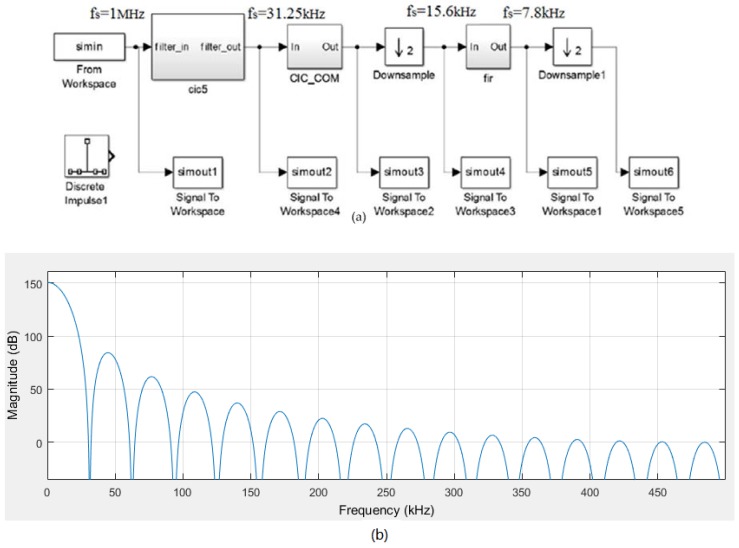

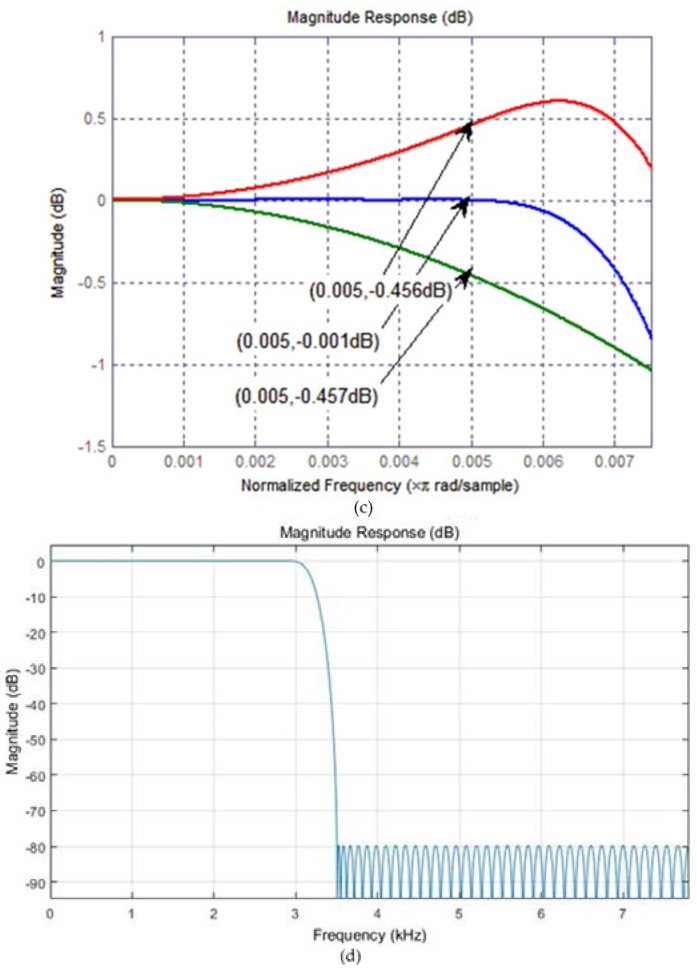
(**a**) Digital decimation filter model (**b**) Frequency response of first-stage cascade integral comb (CIC) filter (**c**) Passband amplitude response of CIC before and after compensation (**d**) Amplitude response of finite impulse response (FIR) low-pass filter.

In view of the design of Sigma-Delta ADC post-stage digital filter, the three stages cascade structure (the CIC filter, CIC compensation, and finite impulse response (FIR) low-pass filter) is selected. After oversampling and noise shaping, the digital signal should be sampling down and filtered. The sampling frequency is reduced to Nyquist frequency. The three stages cascade circuit structure is applied in the filter. The first-stage uses a cascade integral comb (CIC) filter for primary filtering and reducing to a factor of 32 desample frequency; the second-stage uses a CIC compensation filter for ripple compensation and reducing to a factor of 2 desample frequency; the third-stage uses a FIR Half-band filter for high-frequency noise filtering and reducing to a factor of 2 desample frequency. For maximum linearity, it employs a single-bit feedback DAC. The digital signal process chip STM32F405DSC (STMicroelectronicsis Company, Geneva, Switzerland) used for the temperature and linearity compensation. The data transmission to upper computer is achieved by MAX232ESE (Maxim Integrated Products, San Jose, CA, USA). The interface communication mode is SPI and RS232. The 4th-order topology structure is applied in the front-stage Sigma-Delta modulator, the first-stage CIC filter cascading number should be *L +* 1 [36,37,38]. The digital decimation filter model is as shown in Figure 9a. The frequency response of the first-stage CIC filter is shown in Figure 9b. From the diagram, we can see that the 5th-order cascade CIC filter can achieve a sidelobe amplitude attenuation of 70 dB and effectively suppresses the out noise of frequency band. Figure 9c shows the CIC compensation filter (decimation factor *R* = 32, differential delay factor *M =* 1*, L =* 5) cascaded band-pass amplitude response, we can be get that at the normalized frequency *w* = 0.005π, the amplitude can achieve −0.457 dB and −0.001 dB before and after compensation, respectively. The CIC compensation filter can flat the pass-band amplitude and make the transition zone become narrower. In order to further suppress high frequency noise, the FIR filter should have a narrow transition band. The FIR half-band filter has a sampling frequency of 15.6 kHz; a pass-band cut-off frequency of 3 kHz; a stop-band cut-off frequency of 3.5 kHz, and a pass-band attenuation of 0.001 dB. Figure 9d shows the amplitude frequency response of the half-band filter. The digital signal processing modulation is based on micro-processor software programming, so that the digital decimation filter is realized. The function verification software compiler code is transplanted to the micro-processor.

### 3.6. The Test of Three-Axis Digital TMR Magnetometer

**Figure 10:**
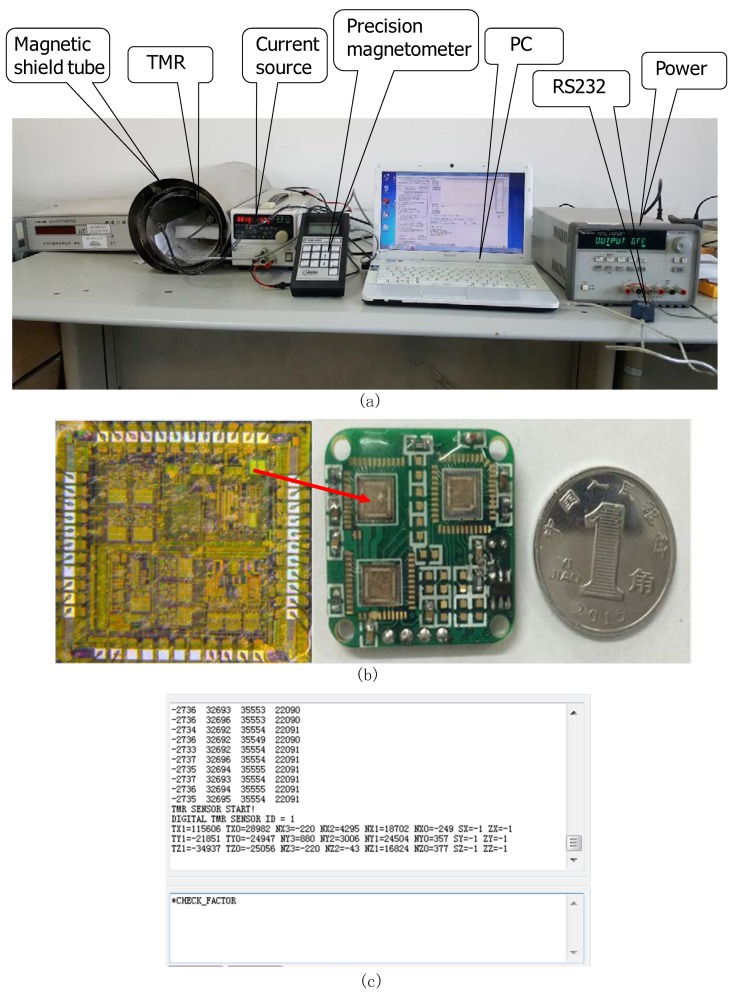
(**a**) The test platform of the three-axis digital magnetic sensor system (**b**) The photograph of printed circuit board (PCB) and ASIC chip (**c**) The data collection by RS232 in PC.

The test platform of the three-axis digital TMR magnetic sensor system is shown in Figure 10a. The interface circuit of the TMR sensor system was implemented in a standard 0.35 μm CMOS process. The printed circuit board (PCB) and ASIC photograph of the tunneling magneto-resistive sensor interface chip is shown in Figure 10b. The aluminum house is used for avoiding magnetic interference. The size of the digital TMR magnetic sensor system is only 25 × 25 × 10 mm^3^. The 5 V power supply is supported by the Agilent E3631 and the power dissipation of the TMR sensor system is 120 mW. The magnetic field is adjustable by the constant-current source Kenwood PW36-1.5ADP (Kenwood Electronics Company, Akaho, Japan). The three-axis fluxgate magnetometer FVM-400 (MEDA Company High-resolution fluxgate, Solna Municipality, Sweden) is useful for measuring the value of magnetic field. The whole test is in a three-layer shield environmental, as shown in Figure 10a. The magnetic signal is shown on personal computer (PC) by RS232 so that we can test the bias drift, linearity and noise performance of the TMR sensor system. The bias drift test of the TMR sensor system is at a zero-magnetic field environmental. The output data of the TMR sensor system is collected by PC. The TMR sensor system can achieve a bias error of 0.01% FS by standard deviation calculation.

**Figure 11:**
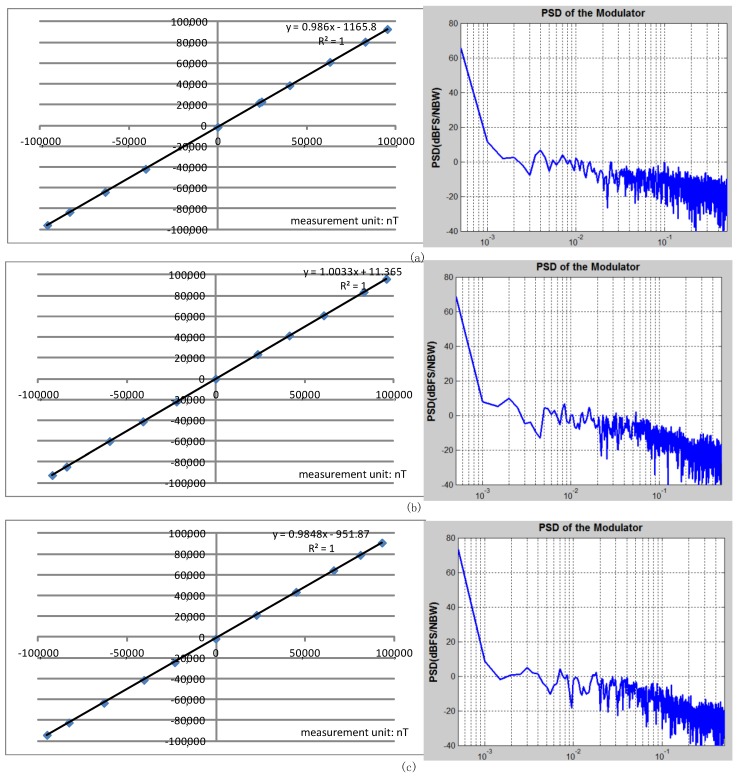
(**a**) The linearity and noise test of TMR magnetometer *x*-axis (**b**) The linearity and noise test of TMR magnetometer *y*-axis (**c**) The linearity and noise test of TMR magnetometer *z*-axis.

The test of the three-axis linearity is as shown in Figure 11 by the fitting straight line at ±1 Guass full scale (1 Guass = 10^5^ nT). The test results of the three-axis linearity are *x*-axis 0.13% FS, *y*-axis 0.11% FS, *z*-axis 0.15% FS, respectively. The PSD of the TMR magnetometer is processed by a standard Matlab program. The TMR magnetometer system achieves a noise density of *x*-axis 250 pT/Hz^1/2^ (at 1 Hz), *y*-axis 240 pT/Hz^1/2^ (at 1 Hz), *z*-axis 240 pT/Hz^1/2^ (at 1 Hz) respectively, which is limited by the low-frequency noise of the TMR sensor element.

## 4. Conclusions

We proposed a high-precision miniaturized three-axis digital tunneling magnetic resistance-type sensor for nanosatellites’ space application. The vacuum-packaged sensitive element by flux chopper can achieve a low background noise of less than 150 pT/Hz^1/2^ (at 1 Hz). The interface circuit is implemented by a standard 0.35 μm 5 V CMOS process. The measurement results show that the TMR sensor system can achieve a better performance as below: at 5 V operating voltage, the sensor system can achieve a power consumption of 120 mW, a full scale of ±1 Guass, a bias error of 0.01% FS, a nonlinearity of *x*-axis 0.13% FS, *y*-axis 0.11% FS, *z*-axis 0.15% FS, and a noise density of *x*-axis 250 pT/Hz^1/2^ (at 1 Hz), *y*-axis 240 pT/Hz^1/2^ (at 1 Hz), *z*-axis 250 pT/Hz^1/2^ (at 1 Hz), respectively. The performance of the TMR sensor element and interface circuit are both beyond Honeywell HMR2300. This magnetic sensor system satisfies the performance requirements in nanosatellites’ space application.

As shown in Table 1, this work based on the TMR sensitive structure is compared with other miniaturized magneto-resistive type magnetometers. Honeywell’s HMR2300 based on the AMR sensitive structure is one of the most widely used in the U.S. GMR50 and G93 are the results of the new research and development in 2017 based on GMR sensitive structure, which have great reference value. Through the comparison of these magnetometers, the TMR magnetometer in this paper has the advantages of low noise, low power consumption and high linearity. The technical index of comprehensive performance can reach a certain level.

## Figures and Tables

**Table 1:** Comparison of this work with other magneto-resistive magnetometers.

Miniaturized Magnetometer	Digital Output	Power/Range (V/Guass)	Consumption (mW)	Noliearity (%FS)	Noise Level (nT/Hz^1/2^)	Sensitivity Axis
HMR2300	Yes	12 V/±1–2 G	400	0.1–0.5	6.67	3
GMR50	Yes	5 V/±1–4.5 G	200	0.5	10–50	1
G93	Yes	5 V/±1 G	380	0.5	10	3
This work	Yes	5 V/±1 G	120	0.11	0.25	3
